# (Whole-Body) Electromyostimulation, Muscle Damage, and Immune System: A Mini Review

**DOI:** 10.3389/fphys.2019.01461

**Published:** 2019-11-29

**Authors:** Marc Teschler, Frank C. Mooren

**Affiliations:** ^1^Department of Rehabilitation Sciences, Faculty of Health, University of Witten/Herdecke, Witten, Germany; ^2^Klinik Königsfeld der DRV, Department of Cardiology and Orthopedics Clinic, Center for Medical Rehabilitation, Ennepetal, Germany

**Keywords:** whole-body electromyostimulation, eccentric, cytokine, training, exercise-induced muscle damage

## Abstract

Exercise-induced muscular damage (EIMD) is a well-known phenomenon in exercise medicine that is closely related to the type and intensity of training, with especially eccentric training content providing various physiological irritations, including mechanical as well as metabolic. Besides the increase in markers of muscular damage, such as creatine kinase (CK) and myoglobin (Mb), several physiological shifts trigger a kind of stepwise repair chain reactions lasting over a time course from several hours to days. Subsequent inflammatory processes are closely related to muscular damage with decisive influence on physiological repair mechanisms, as indicated by an increased invasion of immune cells and typical patterns of pro- and anti-inflammatory cytokines. Previously, whole-body electromyostimulation (WB-EMS) showed significant, partly extreme distractions in markers of muscular damage lasting over several days. Because of the large area of stimulated muscle mass and a relatively high proportion of eccentric movements, initially too intense WB-EMS is predisposed to produce serious changes on several physiological levels due to its unfamiliar muscular strain. Therefore, it is the aim of this short review to focus on the possible immunological side effects of this aspiring training technology. As the number of original investigations in this field is rather small, we will include data from other studies about the relation of exercise-induced muscle damage and immune regulation.

## Introduction

Recently, whole-body electromyostimulation (WB-EMS) has shown to be an effective tool in order to improve muscle strength outcome measurements in deconditioned subjects (Kemmler et al., 2016b, 2017). However, a too intense initial application has been accompanied by some severe side effects. A few case reports have characterized substantial muscle damage and rhabdomyolysis following just one single training session of WB-EMS (Kastner et al., 2014; Finsterer and Stollberger, 2015; Hong et al., 2016; Herzog et al., 2017). In fact, recent studies showed an extreme increase of markers of muscle damage, such as creatine kinase (CK) and myoglobin (Mb) (Teschler et al., 2016; Kemmler et al., 2016a). Symptoms and loss of function during the next hours respectively days following these peak values involved the usual conditions of exercise-induced muscle damage: loss of muscle strength and power, delayed onset of muscle soreness, swelling, reduced range of motion and systemic increases of myocellular enzymes and proteins, or a combination of these (Hyldahl and Hubal, 2014). Frequently, these symptoms last for at least up to 72 h, depending on the volume of muscle-damaging exercise and the extent of disruption of subcellular structures (Fatouros and Jamurtas, 2016).

Such a destruction of tissue and cellular structure is answered by an inflammatory process. This involves a cascade of physiological processes presenting inflammation as a complex interaction of cellular signals and responses (Scott et al., 2004) which initiate subsequent tissue repair and remodeling. The recovering process is dependent on a fine tenement and communication of different cell types such as inflammatory cells (e.g., neutrophils, macrophages); satellite cells (muscle stem cells); vascular cells (e.g., pericytes); and stromal cells (e.g., fibroblasts) (Peake et al., 2017).

The aim of this mini review is (1) to characterize and summarize the basics and key elements of (WB-)EMS, (2) to explore exercise-induced muscle damage and its impact on parameters of the immune system (3) in order to derive conclusions for a reasonable WB-EMS application to avoid negative physiological effects with a special view on immunological effects.

In order to adequately get to the bottom of these questions, we use articles published on Pubmed with relevance to keywords, muscle damage, eccentric, training, exercise, whole-body, electromyostimulation, WB-EMS, cytokine, immunology.

## Basics of Electromyostimulation

The terms electromyostimulation or electrical muscular stimulation (EMS) describe a non-invasive option to stimulate and amplify voluntary muscular contractions. For decades, the use of artificial muscle contractions has been used by physical therapist during rehabilitation processes to postoperatively preserve individual, mostly isolated local muscle groups. Furthermore, EMS is used in the treatment of sports injuries (Lake, 1992), post exercise recovery (Babault et al., 2011; Nedelec et al., 2013) as well as improving athletic performance (Maffiuletti et al., 2000, 2007; Malatesta et al., 2003; Brocherie et al., 2005; Herrero et al., 2005; Babault et al., 2007; Billot et al., 2010).

For more detailed information on local EMS, see Dehail et al. (2008) and Filipovic et al. (2011, 2012).

### Whole-Body Electromyostimulation

**Figure 1:**
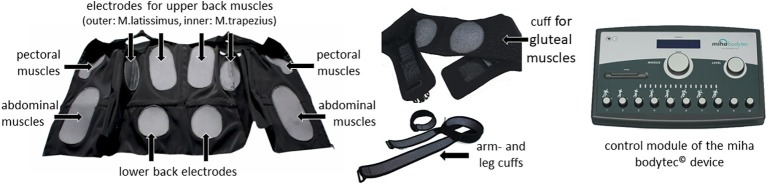
miha bodytec© WB-EMS equipment; from left to right: vest; cuffs for glute, arm, and legs; control device (Permissions were obtained from the copyright holders of the miha bodytec^©^ product for the use for research purposes).

Basically, WB-EMS uses the same physical principle as local electromyostimulation. However, it addresses large muscle groups across the whole body. Instead of reaching just one muscle group, WB-EMS stimulates a muscle area up to 2,800 cm^2^ (Kemmler and von Stengel, 2013) and allows the optional simultaneous stimulation of different muscle groups usually involving the chest, the abs, and back muscles as well as arms, buttocks, and thighs (see Figure 1).

*Via* control module, each single muscle group can separately be controlled, a necessity, as each muscle group has a different impulse sensitivity toward impulse intensity, but also to pain. This depends on the muscular surface and insulation of the muscle by body fat, but also on different pre-activated “starting” positions during various movement patterns performed.

A common training protocol of WB-EMS consists of ~1.5 training sessions per week, each lasting just ~20 min. Despite the low expenditure of net-training time, several studies show significant positive effects on parameters of body composition, especially body fat reduction, strength and power abilities (Kemmler et al., 2010, 2015b,c, 2018; Filipovic et al., 2012). Based on these investigations, the use of a bipolar low-frequency protocol (frequency: 85 Hz, pulse width: 350 μs) is considered as effective for optimal health and training purposes.

Almost all WB-EMS studies use an electrical load ratio of 4–6 s of current vs. 4 s of rest interval (Filipovic et al., 2012; Kemmler et al., 2015b), meaning 10–12 min under load with 8–10 min of rest. The current phase usually characterizes the start of one single repetition, seen in a slow eccentric but joint-friendly and functional full-body movement pattern in manageable range of motion, which makes the training also accessible for people with orthopedic limitations. The rest interval (without current) usually represents the concentric part of the movement to complete the repetition, meaning the (quick) return to the original starting position to recover. An exemplary selection of exercises is shown in Table 1. One training session is therefore defined by the load ratio, while the participants usually go through 1–2 sets with 8–10 exercises and 8–10 repetitions each (Kemmler et al., 2015b,c).

**Table 1:** Exemplary selection of exercises (1–5) performed during WB-EMS with a detailed description of the individual phases of one single repetition (eccentric and concentric).

	Current interval (eccentric)	Non-current interval (concentric)
1.	Squat (4–6 s) and vertical chest press	Squat (4 s up) and vertical rowing
2.	Squat (4–6 s) and latissimus pulldown	Squat (4 s up) with military press
3.	Lunge (4–6 s) with arm-rowing	Lunge (4 s up) with chest press
4.	Squat (4–6 s), crunch with butterfly	Squat (4 s up) and reverse fly
5.	Squat (4–6 s) and trunk flexion (crunch)	Squat (4 s up) and trunk extension

### Treatment With Whole-Body Electromyostimulation

Besides the evaluated positive effects of WB-EMS, some articles report of negative side effects and simultaneously show that EMS is independent of training status (Kastner et al., 2014; Finsterer and Stollberger, 2015; Hong et al., 2016; Herzog et al., 2017). In fact, conducted studies confirmed the symptoms of WB-EMS-induced muscle damage reflected by increases in serum creatine kinase (CK) (Kemmler et al., 2015a; Teschler et al., 2016). Depending on subject groups and their individual training regime, different levels of intensity have been applied. Thus, CK levels after WB-EMS training can range from 1,000 up to 240,000 U/L (Fritzsche et al., 2010; Wahl et al., 2012, 2015; Kastner et al., 2014; Finsterer and Stollberger, 2015; Wirtz et al., 2015; Kemmler et al., 2015a; Filipovic et al., 2016; Hong et al., 2016; Teschler et al., 2016; Herzog et al., 2017) peaking from 72 to 96 h post-exercise (Kemmler et al., 2015a; Teschler et al., 2016). Especially the top levels of this CK range may indicate muscle cell necrosis or severe tissue damage respectively rhabdomyolysis (Baird et al., 2012), which usually cannot be achieved by voluntary training (Yu et al., 2013).

Besides the large volume of muscle mentioned above, and the repetitive activation pattern of the same muscle fibers (Gregory and Bickel, 2005), the underlying decisive but avoidable error in dealing with WB-EMS is the combination of two aspects: (1) type of contraction: WB-EMS uses an increased ratio of eccentric muscular load during each single repetition; and (2) intensity of stimulus: the WB-EMS application may be too intense for the often unaccustomed user. Regarding the practical application of WB-EMS, the focus is usually on the current phase, in which the eccentric part of one single repetition is carried out. Since eccentric workouts are known for higher muscle damage (see section “Exercise-Induced Muscle Damage”), the combination with very intense additional stimulation can especially harm novices more severely.

This misuse of initially intense WB-EMS exposure with corresponding possible severe medical/health-related side effects is meanwhile well examined (Kemmler et al., 2015a; Teschler et al., 2016) and has led to the development of safety guidelines for training with WB-EMS (Kemmler et al., 2016a). Comparable to conventional strength training programs, the guidelines recommend a reduced initial WB-EMS load and the necessity to carefully increase intensity over time. WB-EMS follows the same muscular adaptation principles as seen after conventional strength training protocols. Aldayel et al. (2010) showed a significant reduction of markers of muscle damage already after a second bout of stimulation, as a recent 10-week intervention (1 intense WB-EMS session/week) ultimately showed only moderate enhanced CK levels even after one final WB-EMS session to exhaustion (Kemmler et al., 2015a; Teschler et al., 2016).

## Exercise-Induced Muscle Damage and Its Physiological Consequences

### Exercise-Induced Muscle Damage

The phenomenon of exercise-induced muscle damage (EIMD) is based on an unaccustomed exercise load in relation to the individual’s training status. Thereby, the exercise load may be too intense and/or too frequent and can be followed by a set of symptoms (Paulsen et al., 2012) such as force loss, pain, and stiffness. Those are summarized as the delayed onset of muscle soreness (DOMS), which is biochemically characterized by the release of muscle proteins such as CK and Mb into the circulation (Hyldahl and Hubal, 2014). Tissue histology shows microtrauma to the muscle fiber with the characteristic loss of sarcomere structure such as z-line streaming. Their time courses vary due to the expression of EIMD. The severity of injured tissue may vary from the release of affected macromolecules to large tears in z-disks, sarcolemma, and supportive connective tissue, and induce injury to contractile elements and the cytoskeleton (Paulsen et al., 2012; Koch et al., 2014). Often there is no clear relation between clinical symptoms and the degree of tissue injury.

There is still a discussion whether any kind of muscle action (concentric, eccentric, static) can cause muscular damage (Clarkson et al., 1986; Lavender and Nosaka, 2006; Koch et al., 2014). According to literature, lengthening contractions during slow eccentric movements cause the greatest mechanical strain and consecutive myofibrillar disruption (Newham et al., 1983; Gibala et al., 2000; Lee and Clarkson, 2003; Lavender and Nosaka, 2006; Paulsen et al., 2012; Peake et al., 2017). Different approaches define muscle damage by assessing the change in force development and histological observations. However, the correlation between these parameters often is not very high. The transient ultrastructural myofibrillar disorders are followed by an efflux of myocellular enzymes and proteins, like CK and Mb into the circulation (Peake et al., 2017). This happens in close relation to duration, intensity, and type of muscular activity (Stein, 1998; Howatson et al., 2005). Although CK levels show a high interindividual variability, their high sensitivity makes CK a considerable marker of severe muscle cell damage, muscle cell disruption, or disease (Brancaccio et al., 2008; Baird et al., 2012).

Mild to moderate EIMD is defined by a force reduction by 20–50% of the one-repetition-maximum (1RM) with total recovery in between 2 and 5 days and CK levels below 10,000 U/L. Pronounced manifestations are characterized by reduced force-capacity above 50% 1RM with serum CK levels rising above 10,000 U/L (Paulsen et al., 2012) including a total recovery phase of longer than 7 days. This range of severe EIMD includes the criteria for a clinically relevant rhabdomyolysis, which is defined as a 50-fold rise in serum CK and muscle necrotic symptoms like pain/tenderness, swelling, weakness, and myoglobinuria (Visweswaran and Guntupalli, 1999; Paulsen et al., 2012; Zutt et al., 2014). Finally, muscle damage is followed by an inflammatory response including cellular infiltration, phagocytosis, and cytokine release (Newham and Jones, 2016).

### Exercise-Induced Muscle Damage, Inflammation, and Regeneration

The link between exercise, muscle damage, and inflammatory processes after (eccentric) exercise has been extensively investigated (Paulsen et al., 2012; Hyldahl and Hubal, 2014; Chazaud, 2016). Exercise is a well-accepted modulator of immune cell count and function (Simpson et al., 2015), seen in its capability to mobilize leucocytes, especially neutrophils, into the circulation and prolongate their lifespan *via* different hormonal pathways (Mooren et al., 2012). Meanwhile there is an increased acceptance that the initiated inflammatory process is a mandatory key for muscle repair and regeneration (Chazaud, 2016). Exercise-associated alterations of the hormonal environment such as the catecholamines, adrenaline and noradrenalin, and/or cortisol are important in activating and modulating several types of immune cells (Koch, 2010). Several physiological processes lead to an adaptive remodeling and a renewed homeostasis (Chazaud, 2016) of muscle and connective tissue including the extracellular matrix (ECM) (Mackey and Kjaer, 2017).

The initial physiological stress causes mechanical damage seen in microtrauma in myofibers followed by an invasion of pro-inflammatory macrophages. An early release of further inflammatory mediators leads to vasodilation including subsequent increase in vascular permeability (Sass et al., 2018). This affects injured muscle cells and the adjacent connective tissue leading to tissue swelling and muscle stiffness. Additionally, the training stimulus causes metabolic irritations (e.g., temperature, reduced mitochondrial respiration, lowered pH value, reactive oxygen production). The progressive loss of Ca^2+^ homeostasis happens due to the release from either leaky intracellular stores or the influx across leaky plasma membrane. Such increases of intracellular Ca^2+^ activate Ca^2+^-dependent proteases such as calpain that is known to degrade contractile proteins and/or excitation-contraction coupling proteins, which is supposed to be one explanation for prolonged force reduction (Kendall and Eston, 2002; Hyldahl and Hubal, 2014; Sass et al., 2018).

Increased intracellular Ca^2+^ causes damage on myofilaments of skeletal muscle (Bingham et al., 1997) also through activation of phospholipase A_2_. The induced injury to the sarcolemma is supported by the production of leukotrienes and prostaglandins through free reactive oxygen species (ROS) and/or release of detergent-like lysophospholipids (Armstrong, 1990; Kendall and Eston, 2002). Permeable membrane conditions favor a further efflux of intracellular lysosomal enzymes (Armstrong, 1990; Byrd, 1992; Clarkson and Sayers, 1999).

The post-exercise-initiated regeneration process of muscle tissue is characterized by a stepwise invasion and activation of different types of immune cells. Initially, the muscular cell composition is altered by an evenly doubled number of neutrophils and lymphocytes, a small contribution of monocytes (Koch, 2010), and a 10-fold increase of natural killer cells (Campbell and Turner, 2018). Thereby, the acute and persistent sympathetic nervous activity with increased number of catecholamines plays an important role in recruiting neutrophils and lymphocytes, especially T- and B-cells (β2) *via* expressed α- and β-adrenergic receptors (Peake et al., 2017). In contrast, the post-exercise period is characterized by alternations of an exercise volume-dependent lymphopenia (Kruger and Mooren, 2007).

Neutrophils directly migrate into the inflamed area and start to remove cell debris (phagocytosis). After leaving the circulation, monocytes enter the injured tissue and transform into macrophages (Koch, 2010). These interactions define the first line of defense though increased numbers of neutrophils and pro-inflammatory macrophages (M1) contribute to further muscle injury and further impairment of muscle remodeling and functional recovery due to high cytotoxicity and capacity to lyse muscle cells (Peake et al., 2017).

The high potential of phagocytic cells releases additional ROS with a high oxidizing effect on fats, proteins, nucleic acid, and ECM. These metabolic processes support the inflammatory response and progressive cell damage by promoting the expression of pro-inflammatory cytokines (Pyne, 1994; Best et al., 1999). The alternations in muscle tissue integrity are accompanied by impairments of oxygen supply. This results in a hypoxic atmosphere with decreasing pH value. Here, just certain immune cells are capable to switch to anaerobic metabolism, to survive and to consequently trigger the necessary inflammatory response by cytokines and chemokines (Sass et al., 2018). Lymphocytic B-cells interact with T-helper cells, whereas lymphocytic T-cells accelerate the immune response through cytokine secretion (Koch, 2010). Mediating the communication between different types of cells such as immune, muscle, and ECM cells, the production of cytokines is one of the most important responses to exercise.

Early post-exercise hours (4–24 h) of muscle damage are typified by pro-inflammatory macrophages (M1); extended release of pro-inflammatory cytokines like interleukin-1β (IL-1β), tumor necrosis factor-α (TNF-α) and IL-6; and initiation of myoblast proliferation. Depending on tissue environment, the dynamic phenotype plasticity of macrophages allows a shift from pro-inflammatory (M1) to anti-inflammatory (M2) status – this presents a central part of resolution of an inflammation, influenced by phagocytosis, IL-10 and AMP-activated protein kinase-alpha (AMPK-α). This first step of regeneration attenuates inflammation through production of anti-inflammatory cytokines like IL-10, tumor growth factor-β1 (TGF-β1), and insulin-like growth factor (IFG-1) (Peake et al., 2017).

Twenty-four hours post EIMD, the rise in anti-inflammatory macrophages (M2), CD8- and T-regulating lymphocytes promotes further anti-inflammatory cytokines and macrophages, increasing myoblast and satellite cell (SC) proliferation. The activation of stromal cells (fibro-adipogenic progenitors, pericytes) supports the myoblast differentiation. Muscle regeneration as a process of increased muscle protein synthesis is characterized by increased numbers of SC that subsequently proliferate, differentiate, and enter damaged myofibers either to heal fibers or to synthesize new fibers (Fatouros and Jamurtas, 2016). In this regard, Crameri et al. (2007) found histological evidence for significant increased numbers of myofiber proteins and SC markers in electrically stimulated leg muscles compared to voluntarily trained ones. The invasion of macrophages, which contribute to SC proliferation, their differentiation into myoblasts, and forming of new myotubes, thus presents the necessary prerequisite for muscle regeneration (Kendall and Eston, 2002).

As seen, exercise is a potent effector of several leucocyte functions such as oxidative burst, phagocytosis and, with increasing importance, the expression of cytokines. The role of cytokines in intercellular signaling processes has been rewritten by findings that show that tissues besides the classical immunological tissues are able to release cytokines (Munoz-Canoves et al., 2013). As muscle tissue has been shown to be a major source of interleukin-6 (IL-6) during exercise, these most prominent alternations led to the acceptance of IL-6 as myokine.

The effect of EMS on exercise-induced alternations of the immune phenotype, immune cell count, or immune function has rarely been investigated. Two studies investigated the effect of superimposed EMS during cycling on IL-6 and brain-derived neurotrophic factor (BDNF) (Wahl et al., 2015) respectively on IL-6 and human growth hormone (GH) (Omoto et al., 2015). While EMS application was followed by a substantial release of muscle damage markers such as CK and Mb, the effect on the myokines was only marginal (Omoto et al., 2015; Wahl et al., 2015). Likewise, no effects of EMS on strength training-induced mobilization of testosterone, cortisol, and human growth hormone could be observed (Wirtz et al., 2015). On the other hand, one single bout of isometric electrical stimulation found significantly greater muscle damage and GH (Jubeau et al., 2008) compared to voluntary exercise.

## Conclusion, Recommendations, and Practical Considerations for (Whole-Body) Electromyostimulation

As the literature concerning (WB-)EMS is rather manageable, the few presented studies additionally provide a high inconsistency in the application (dynamic vs. isometric, frequency, load ratio, different electrodes). Thus, in addition to pronounced ranges of muscle damage, there is currently insufficient knowledge about the effect of (WB-)EMS on immunological parameters to report.

The abovementioned evidence of EIMD, seen in, e.g., CK, after a very intense application of (WB-)EMS shows that the technique must be handled with a certain responsibility and special care. As seen, a misapplication may also be followed by immunological deflections; however, in this regard detailed investigations are still lacking.

By now, there is no evidence for severe immunological alternations after WB-EMS. But the meanwhile widespread use of WB-EMS in general and especially the growing interest for implementation of (WB-)EMS training in clinical and rehabilitation settings require even more detailed evidence about further underlying mechanisms and physiological side effects. To ailing patients, knowledge about acute and prolonged immunological effects would be useful and essential. Closing this gap should be one objective of further research to guarantee an even safer application of WB-EMS in the future.

Nevertheless, the influence of EMS on muscle damage and immunological findings or deflections is quite manageable if the technology is used correctly.

## Author Contributions

MT and FM revised the literature and wrote, revised, and approved the manuscript.

### Conflict of Interest

The authors declare that the research was conducted in the absence of any commercial or financial relationships that could be construed as a potential conflict of interest.
